# Knowledge, Attitudes, and Risk Perception Toward Avian Influenza Virus Exposure Among Cuban Hunters

**DOI:** 10.3389/fpubh.2021.644786

**Published:** 2021-07-23

**Authors:** Beatriz Delgado-Hernández, Lourdes Mugica, Martin Acosta, Frank Pérez, Damarys de las Nieves Montano, Yandy Abreu, Joel Ayala, María Irian Percedo, Pastor Alfonso

**Affiliations:** ^1^Epidemiology Group, National Center for Animal and Plant Health (CENSA), World Organisation for Animal Health (OIE) Collaborating Center for the Reduction of the Risk of Disaster in Animal Health, San José de las Lajas, Cuba; ^2^Bird Ecology Group, Biology Faculty, Havana University, Vedado, Cuba; ^3^Department of Veterinary Medicine, Faculty of Agricultural Sciences, University of Granma, Bayamo, Cuba

**Keywords:** avian influenza, hunter, wild bird, risk perception, pandemic, One Health, Cuba

## Abstract

A critical step for decreasing zoonotic disease threats is to have a good understanding of the associated risks. Hunters frequently handle potentially infected birds, so they are more at risk of being exposed to zoonotic avian pathogens, including avian influenza viruses (AIVs). The objective of the current study was to gain a better understanding of Cuban hunters' general hunting practices, focusing on their knowledge and risk perception on avian influenza. An anonymous and voluntary semi-structured questionnaire was designed and applied to 398 hunters. Multiple correspondence analyses found relationships with potential exposure of AIVs to people and domestic animals. The main associated risks factors identified were not taking the annual flu vaccine (60.1%) and not cleaning hunting knives (26.3%); Direct contact with water (32.1%), cleaning wild birds at home (33.2%); receiving assistance during bird cleaning (41.9%), keeping poultry at home (56.5%) and feeding domestic animals with wild bird leftovers (30.3%) were also identified as significant risk factors. The lack of use of some protective measures reported by hunters had no relationship with their awareness on avian influenza, which may imply a lack of such knowledge. The results evidenced that more effective risk communication strategies about the consequences of AIVs infecting human or other animals, and the importance of reducing such risks, are urgently needed.

## Introduction

Influenza A viruses (IAVs) are among the most challenging viruses that threaten both human and animal health (1). Their ability to transmit between different species and, to undergo genetic reassortments is extremely alarming (2). In fact, the best studied pandemic influenza viruses, like those of 1918, 1957, 1968, and 2009, ultimately acquired some or all of their gene segments from the avian IAV gene pool with swine origin genes also being present in some viruses (3).

Wild aquatic birds, especially birds in the orders Anseriformes (ducks and geese) and Charadriiformes (gulls and shorebirds) that migrate in large numbers from North America to Cuba, are considered natural hosts for most IAV subtypes (4, 5). In fact, the migratory nature of many waterfowl species, along with the potential persistence of avian influenza viruses (AIVs) in them, presents a potential route for global dissemination and spillover of these viruses (6, 7).

Individuals who are engaged in occupations requiring animal handling (hunting, butchering, etc.) or those working in agricultural areas or forests, are at increased risk of exposure to AIVs compared to the general population (8). Direct transmission of AIVs from wild birds to hunters, or anybody interacting with wildlife, might have at least two significant outcomes; a direct introduction of a novel virus that could be sustained by human-to-human transmission, or a possible reassortment event where avian genes could be incorporated into an existing seasonal human influenza strain (9).

Poultry keepers and wild bird hunters are considered at highest risk of contracting AIV infections (10). However, wild bird hunters are likely to be at highest risk considering the high number of people involved in hunting and the direct nature of their contact with dead wild birds and bird carcasses during cleaning. Furthermore, some duck species which are commonly hunted in Cuba are known to have the highest prevalence level for AIVs (4). Since AIVs are known to replicate in wild bird in the absence of overt signs of disease (11), it is possible that apparently healthy hunted birds could spread AIVs to the hunters.

In addition to the direct AIV exposure risk for hunters, they may also indirectly cause the dispersal of such viruses in the environment, with the possibility of spillover to other species. It is known that AIVs are able to infect a broad range of host species (5) include several mammals and poultry, on occasions with significant economic losses. Despite the fact that AIVs often exist in their wild bird reservoir host as low pathogenic viruses (12), when they infect poultry, they can evolve to cause serious disease termed highly pathogenic avian influenza (HPAI), with severe economic consequences (13). Since the poultry sector provides one of the most popular sources of animal protein around the world, owing to its affordability, nutritional value and lack of cultural restrictions, AIVs represent an important threat to food security. All these facts clearly demonstrate the need to address the associated risks from a “One Health” perspective (14).

Good knowledge, attitudes, and practices (KAPs) targeted toward certain diseases or infections among the public are essential for successful control and outbreak prevention of pandemics (15, 16). However, efforts to better define KAPs in hunters have been scarce, and mainly limited to Canada and the United States of America (17–19). Since, behaviors and risk attitudes can vary from country to country, studies in different countries where different practices are carried out are well-justified. The objective of the current study was to gain a better understanding of Cuban hunters' general harvesting practices, knowledge and risk perception on avian influenza (AI).

## Materials and Methods

### Survey

A semi-structured questionnaire (see Supplementary Material) was designed taking into account related works on this subject (17, 18). The survey was validated by local knowledgeable hunting specialists (*n* = 3) and a small group of local wild bird hunters (*n* = 5). A total of 398 Cuban wild bird hunters were recruited to the study. The survey was conducted opportunistically taking advantage of planned meetings between 2016 and 2018 of the Sport Hunting Cuban Federation (FCCD), which has around 4,025 members (20). No information on avian influenza was provided to hunters before giving them the questionnaire. The surveys that were <50% completed were discarded. For statistical purposes, in the cases of incomplete surveys the proportions of the response were rescaled according to denominators of the completed answers.

### Descriptive Analysis

The demographic variables were analyzed through descriptive statistics. The variables of age and experience of the hunter were categorized according to the median. Variables related with risk or knowledge (Table 1) were compared by proportion analysis with a confidence interval of 95% using the WINPEPI application (21) and a Wald Test in the CompaProp application (22). To evaluate the risk perception level about AI, a univariate and multivariate logistic regression were carried out with *p*-value <0.05 using the SPSS v.21 program. For this analysis, hunters were categorized according to their hierarchic status (Hunters belonging to the FCCD Steering Committee vs. those only dedicated to hunting) within the Federation.

**Table 1:** Variables related with knowledge, perception and risk attitudes used for analyses.

**Variable**	**Kind of variable**	**Description**
Knowledge about avian influenza	Knowledge	As a potential proxy for adopting protective measures
Wild bird hunting as risk for health	Knowledge	As a potential proxy for adopting protective measures
Water contact during hunting	Attitude	As a proxy for getting infection from water
Smoking	Attitude	As a proxy for getting infection through oral way with contaminated hands
Hunting with dogs	Attitude	As a proxy to reduce contact with water
Cleaning hunted birds at home	Attitude	As a proxy for AIV dissemination to new locations
Get assistance for birds cleaning	Attitude	As a proxy to expose additional individuals to virus infection
Sharing hunting knives with household uses	Attitude	As a proxy to contaminate food
Cleaning knives after hunting	Attitude	As a proxy for reducing risk of infection
Having backyard poultry at home	Attitude	As a proxy to propitiate AIV to evolve
Feed domestic animal with bird leftover	Attitude	As a proxy for spillover
Unvaccinated against flu	Protective	As a proxy for virus viral genome reassortments in case of coinfections
Washing hands during hunting	Protective	As a proxy for reducing risk infection

### Multivariate Analysis

Multiple correspondence analysis (MCA) and hierarchical cluster analysis were executed using the *FactoMineR* package (23) through R v3.5. The variables with *p-*values higher than 0.05 were discarded. A minimum number of latent variables (or components) with linear combinations of the original variables that are independent from each other were defined (24). The number of dimensions in the analysis was selected according to the percentage of inertia.

MCA was also used in pre-processing to transform categorical variables into continuous ones in order to perform a cluster analysis by ascending hierarchical classification (Ward's method and Euclidean similarity distance between observations) (24, 25). Homogeneous subject profiles based on the MCA dimensions assuming that they have substantive coherence (24, 26) were defined. The coordinate distribution of MCA categories in a two-dimension space based in eigenvalues and the variable description by categories of clusters were combined for the result representation with Ggplot2 package in R v3.5. The variables: Knowledge about avian influenza, Wild bird hunting as risk for health, Age and Experience Categorized, and Hierarchic status were used as supplementary variables in the MCA and cluster analysis.

#### Ethical Approval

Participants were provided with information describing the study objectives and they were reassured that all responses would be anonymous.

#### Informed Consent

Verbal informed consent of willingness to participate in the study was obtained from each respondent before they filled in the questionnaire.

## Results

A total of 398 out of 403 (98.76%) surveys were valid from which 305 (76.63%) were completed in full. Of the valid surveys 17.83% belonged to hunters with coordination responsibilities at the provincial or national level in the FCCD. Among responders, only one was female. The number of hunting days/year and the number of hunted birds/year accounted for the higher variability in the descriptive analysis (Table 2). Ducks were hunted by 215 out of 252 (85.3%) of the surveyed hunters, of which 82 (38.14%) referred to the capture of Blue-winged teal (*Spatula discors)*.

**Table 2:** Interquartile ranges of quantitative and demographic variables of wild bird hunters surveyed from 2016 to 2018 (Q1: quartile 25%, Q3: quartile 75%, IQR: interquartile range).

**General variables**	**Minimum**	**Q1**	**Median**	**Q3**	**IQR**	**Maximum**
Number of hunting days per year	1	30	40	80.25	50.25	240
Number of hunted birds per year	3	44	61	143	99	1,800
Quantity of hunting months per year	1	5	6	7	2	12
Hunter age	17	41	50	59	18	85
Hunting experience (years)	1	10	17	30	20	82

The categorized variables formed two groups based on age and experience: young people (≤50 years) and older people (>50 years), as well as hunters (≤17 years) with little hunting experience and the most experienced hunters (>17 years).

Six groups were formed according to the proportion of the risk factors. Hygienic practices with knives (85%, CI95%:82–89%) and hands (86%, CI95%:81–88%), Water contact during hunting (77%, CI95%:73–81%), Knowledge about avian influenza (75%, CI95%:71–79%) and cleaning hunted birds at home (74%, CI95%:70–79%) were the questions with a greater proportion with affirmative replies. The questions related to hunter's attitudes (two last groups) had a lower proportion of positive answers (<50%) (Table 3).

**Table 3:** Relationship of risk attitudes and knowledge of Cuban hunters on avian influenza virus exposure.

**Variable**	**Total answer**	**Proportion of affirmative replies (CI 95%)**	**Wald test significance**
Washing hands after hunting	393	0.86 (0.822–0.893)	a
Cleaning knives after hunting	379	0.85 (0.810–0.884)	a
Water contact during hunting	396	0.77 (0.728–0.813)	b
Knowledge about avian influenza	393	0.75 (0.705–0.793)	b
Cleaning hunted birds at home	393	0.74 (0.697–0.786)	b
Flu unvaccinated against flu	393	0.64 (0.594–0.591)	c
Wild bird hunting as risk for health	394	0.59 (0.544–0.643)	c
Hunting with dog	397	0.58 (0.532–0.631)	c
Having backyard birds at home	388	0.48 (0.431–0.533)	d
Get assistance for bird cleaning	397	0.46 (0.414–0.514)	d
Sharing hunting knives with household uses	393	0.46 (0.405–0.506)	d
Feed domestic animal with birds leftover	387	0.38 (0.331–0.430)	e
Smoking	395	0.35 (0.307–0.404)	e

*Proportions with different letters in Wald test differs according to the calculation of confidence intervals*.

*CI, confidence interval*.

Eight out of 13 studied variables were significant (*p* < 0.05) in the univariate analysis (Supplementary Table 1). Of these eight variables, having backyard poultry at home and smoking were significant in the multivariate analysis with an odds ratio (OR) of 2.37 (CI 95%: 1.247–4.515) and 2.203 (CI 95%: 1.083–4.483), respectively, for the hunters with managerial responsibilities with respect to pure hunters (Table 4). However, these categories did not have any significant differences in knowledge on AI.

**Table 4:** Maximum likelihood estimates of multivariate regression function of variables derived from individual analyses between Manager hunters and Pure hunters.

**Variable**	**B**	**SE**	**Wald**	**P**	**Odds ratio**	**95% CI for odds ratio**
Feed domestic animal with birds leftover	0.613	0.336	3.331	0.068	1.846	0.956–3.566
Having backyard poultry at home	0.864	0.328	6.936	0.008	2.373	1.247–4.515
Smoking	0.790	0.362	4.751	0.029	2.203	1.083–4.483
Cleaning hunted birds at home	−0.885	0.446	3.925	0.048	0.413	0.172–0.991
Hunting with dog	−0.896	0.339	6.974	0.008	0.408	0.210–0.794
Knowledge about avian influenza	−1.401	0.548	6.533	0.011	0.246	0.084–0.721

*B, estimated slope; S.E., standard error; Odds ratio: [Exp (B)]*.

### MCA and Hierarchical Cluster Analyses

A variability of 61.9% was observed for the four first dimensions in the MCA analysis of hunters' exposure to AIVs. The variables with the main contribution to the first dimension were: be unvaccinated against flu (60.1%), be a smoker (54.4%) and not cleaning hunting knives (26.3%). The variables of direct contact with water (32.1%) and cleaning wild birds at home (33.2%) headed the second dimension. The third dimension included hunters that received assistance during bird cleaning (41.9%) and hunters who did not wash their hands (23%), while the fourth dimension was represented by people that used hunting knives in household activities (67.8%) and hunted with dogs (33.9%) (Supplementary Table 2).

**Figure 1:**
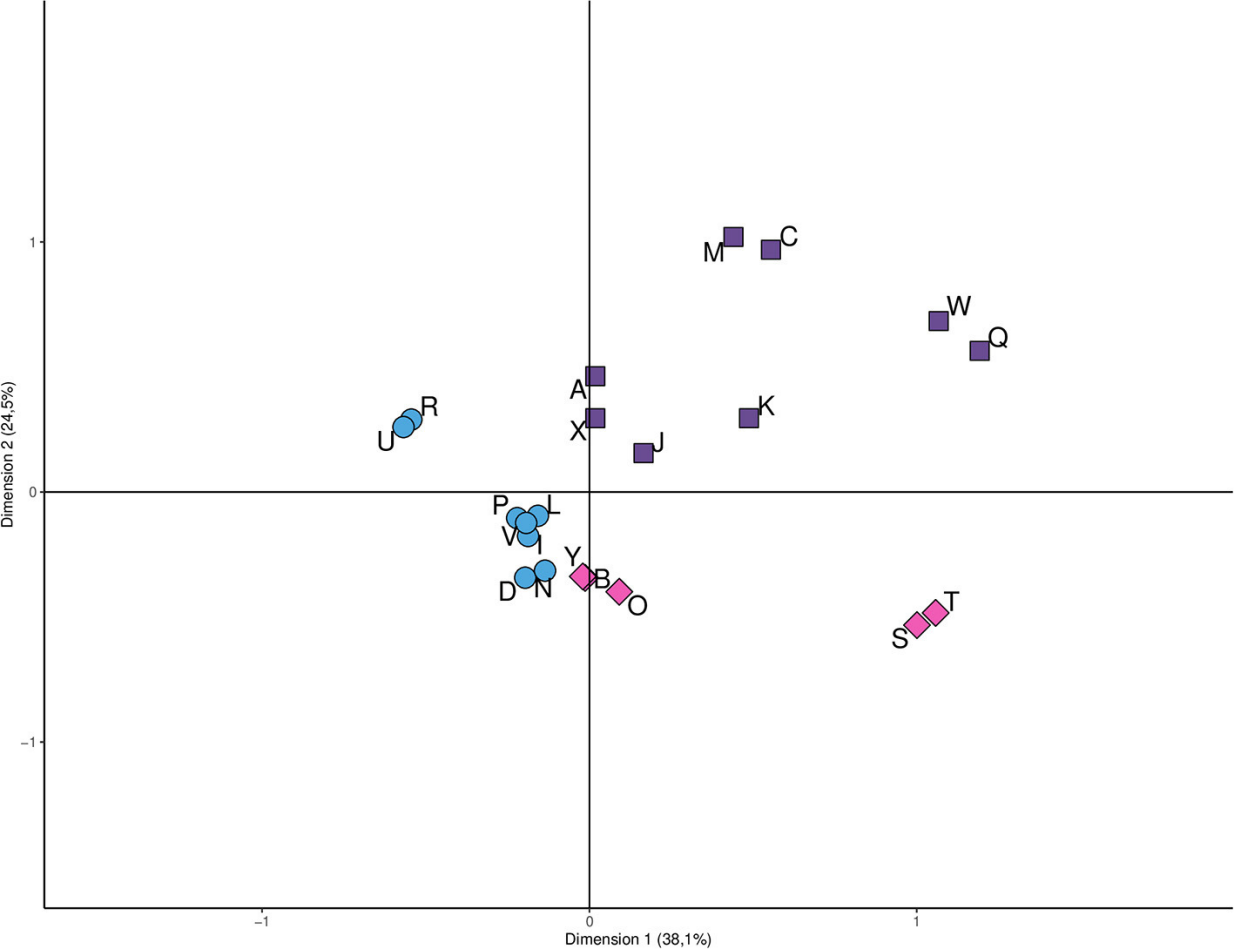
Clustering of variables associated with the potential exposure of hunters to avian influenza viruses. Symbols with the same shape or color belong to the same cluster and their particular characteristics appear below in correspondence with the letter that identifies them. **Cluster 1:** Clean birds at home (D), High hunter experience (I), Have knowledge on avian influenza (L), Direct contact water (N), Clean knives after slaughtering (P), Don't smokes (R), Flu unvaccinated (U), Washing hands after hunt (V); **Cluster 2:** Don't hunt with dog (A); Don't clean birds at home (C), Low hunter experience (J), haven't knowledge on avian influenza (K), Haven't direct contact water (M), Don't clean knives after slaughter (Q), Don't washing hands after hunt (W), Sharing hunting knives in household activities (X); **Cluster 3:** Hunt with Dog (B), Receives bird cleaning assistance (O), Smokes (S), Flu vaccinated (T), Don't share hunt knives in other activities (Y).

The hunter's practices and/or attitudes that exposed them to AIVs were identified into the three groups (Figure 1). Most of the risk categories were within the first cluster whilst the second group was the smallest.

The inertia of two first dimensions was 62.66% for analysis of AIV exposure to domestic animals. The variables hunting with dog (56.4%), have poultry at home (56.5%) and feed domestic animals with bird leftover (30.3%) predominated in the first dimension. The transfer of hunted birds home for cleaning was the most represented variable in the second dimension (83%) (Supplementary Table 3).

**Figure 2:**
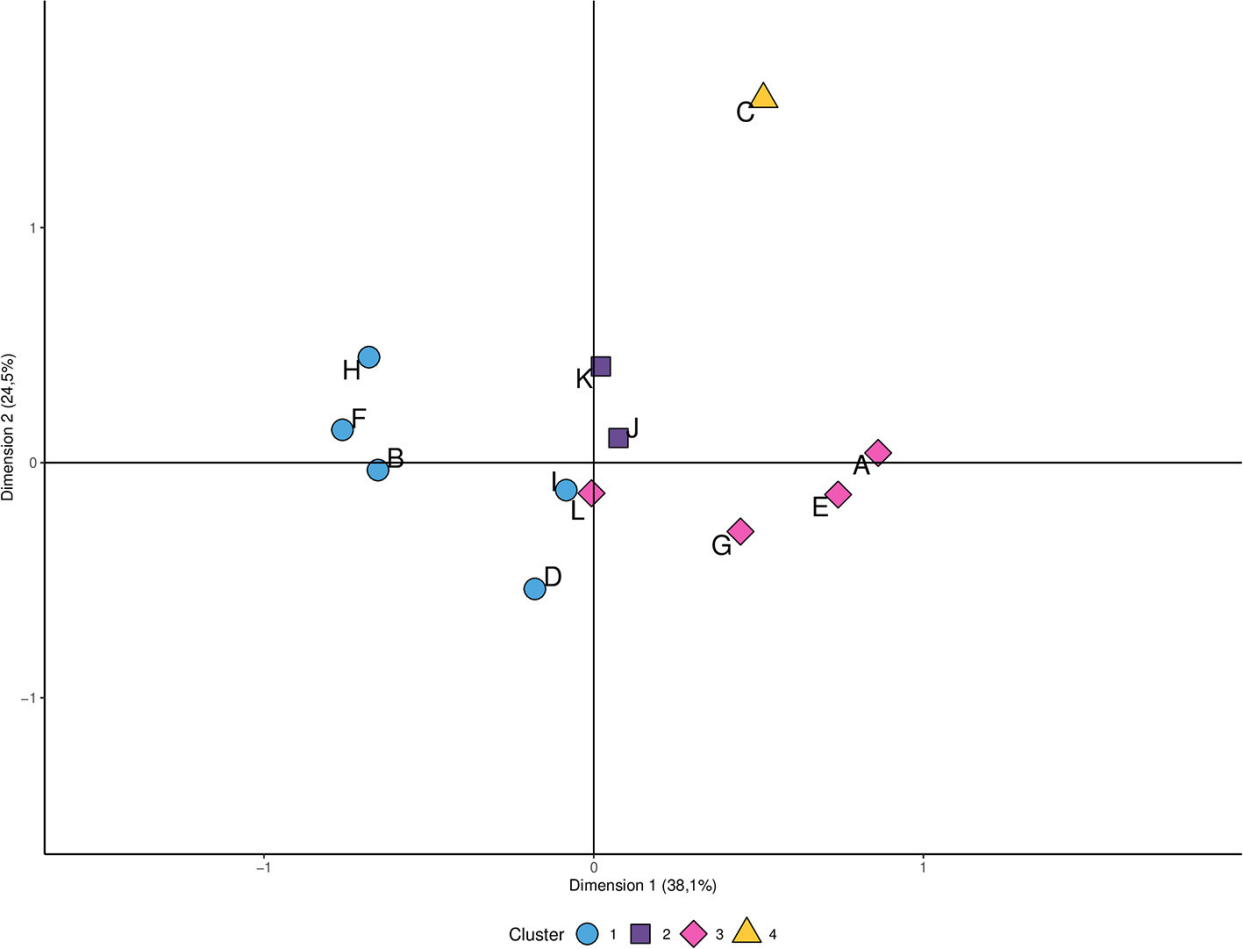
Clustering of variables associated with the potential exposure of domestic animal to avian influenza viruses. Symbols with the same shape or color belong to the same cluster and their particular characteristics appear below in correspondence with the letter that identifies them. **Cluster 1:** Hunt with dog (B); Clean birds at home (D), Having backyard poultry at home (F), Feed domestic animal with bird leftover (H), High hunter experience (I); **Cluster 2:** Low hunter experience (J), Haven't knowledge on avian influenza (K); **Cluster 3:** Don't hunt with dog (A), Haven't backyard poultry at home (E), Don't feed domestic animal with bird leftover (G); Have knowledge on avian influenza (L); **Cluster 4:** Don't clean birds at home (C).

According to hunter's behavior, four groups were obtained in the cluster analysis (Figure 2). Interestingly, hunters who did not clean birds at home didn't share characteristics with any of the other clusters. On the contrary, the first cluster showed a high potential risk of AIV exposure for domestic animals. The variables hunter age and hunting experience were not associated to the other variables.

## Discussion

This study targets the “first-line” people (wild bird hunters) who might both acquire infection with AIVs and expose domestic animals to them. Cuban hunters were found to have limited knowledge of avian influenza and associated risks which demands a more effective risk communication strategy to bridge the gaps between knowledge and practical actions.

The current investigation did not show a relation between knowledge on AI and the adoption of protective measures. Therefore, it is likely that the understanding on AI of the surveyed hunters could be rudimentary or insufficient to be translated into protective behaviors. However, this knowledge was greater in terms of the risk of exposure of AIV to domestic animals. This could be related to the fact that risk communication has been focused on the consequences of infection with AIVs for poultry, compared to infection of human. Another possible explanation for such differences is that effects might depend upon the specific type of knowledge measured (10).

Limited knowledge, low risk perception and inadequate protective behavior can increase the risk of infection with AIVs (10). However, differences between stated knowledge and practical knowledge are recognized (27). Most hunters were aware of AI but were not actively preventing the introduction and transmission of the virus as they perceived it as a low risk to their health, as described by Oruganti et al. (19). Likewise, other investigations show high AI knowledge levels but insufficient adoption of protective measures (10, 28). Just because hunters may know about a wildlife disease and how to prevent exposure to it does not imply they perceive a risk of exposure (19). The fact that knowledge about AI did not translate into protective behaviors was notable even within the subgroup of hunters with organizational responsibilities within the FCCD. This emphasizes the need for risk communication actions with emphasis on those in a position to play a more active role in the transfer of knowledge within the Federation.

Preventive measures such as hand washing and wearing masks are fundamental for counteracting influenza virus infection (29). The data on protective behaviors showed that washing hands was a standard practice. However, accessible water in wetlands may be contaminated with water-borne microorganisms. In particular, a study about the potential for avian-origin viruses to remain infective in North American wetlands for extended periods proved its viability at a mean temperature of 4.2–4.9°C (−0.1–22.9°C) (30). Given the lipid nature of the envelope of IAVs (29, 31) the most practical and effective method of decontamination during hunting is the use of alcohol gels as a disinfectant.

Consistent with other studies (10, 18, 32) washing hands and cleaning hunting utensils after finishing the activity were the most prevalent practices, which can reduce hunter's AIV exposure. Remarkably, inexperienced hunters who don't know about AI, practiced these activities less frequently, which highlight the importance of knowledge.

Knowledge about effective behaviors is particularly likely to enhance perceptions about efficacy of conducts, which have consistently been linked to precautionary practices (33). Nevertheless, knowledge alone is not enough to produce behavior changes because it depends of economic and social factors that enable or disable such change (10). Consequently, effective risk communication strategies could be necessary to improve the knowledge level and generate protective attitudes and practices to reduce exposure risk to AIVs.

The low flu vaccination coverage in the surveyed hunters, may be due to less concern about infection, which is the strongest predictor of vaccination uptake (34) and it constitutes a demand for actions to reduce the risk of reassortment of IAVs in this population stratum. Vaccination is the main measure for preventing seasonal influenza and its potential complications (35). Vaccination of groups with a higher risk of exposure to AIVs, such as poultry workers, is recommended by the anti-pandemic Global Action Plan (36). In Cuba since 1998, the National Vaccination Policy for Seasonal Influenza prioritizes at risk groups (37, 38).

The lack of vaccination in people at higher risk of being exposed to AIVs implies a greater risk of co-infection with different strains, which may lead to reassortment events with potentially harmful consequences. Cross-species transmission of AIVs directly from wild birds to humans is rare, but given the increased risk of exposure to AIV infection in hunters (9), it is clear that they should be prioritized for the seasonal flu vaccination. Evidences of AIV infection in persons with occupational exposure to migratory birds (39) and human coinfection with different AIVs, have been reported (40).

The flu season in American tropics mainly occurs from April to September (41) while long term studies in Cuba, show human influenza virus circulation increases during the rainy season (May-October) (42), which partially overlap with the waterbird migration season during the fall (43). These facts exacerbate the risk of coinfection with IAVs, that are increased in some species of hunted-waterbird with a high prevalence of AIVs like *Spatula discors* (4).

Flu vaccination strengths immunity against human influenza viruses at a population level by reducing the likelihood of coinfection hence decreasing the possibility of generating new progeny viruses by genetic reassortment (44). However, given flu vaccination does not prevent infections by AIVs, other preventive measures must be put in place to complement the reduction of the risk of human infections with AIVs, some of which may cause severe consequences (45).

Wild animal slaughtering, whether done by hunters or their family members, can place both at risk of transmission through direct exposure to blood and internal organs as well as feces (8). Hence, being helped by another family member during bird cleaning, additionally increased the risk of exposing more people. On the other hand, since other family members may be not considered at risk, they may lack protective measures like flu vaccination, and be more prone to IAV coinfection events.

The practice of slaughtering wild birds at home may also increase food safety risks because some pathogens and infectious agents are usually found in meats (46). In particular, the delay in bird processing after hunting may increase infection risks e.g., through the transfer of enterobacteria from gut to muscles resulting in food borne transmission. Since AIV infections can occur through direct contact with tissues, secretions and excretions of infected birds (9) it is necessary to reduce or eradicate the practice of cleaning hunted birds at home, as well as the use of hunting knives for other household activities.

Smoking prevalence among the surveyed hunters was similar to that of the general population in the country (47). However, smoking in addition to important health implications (48), when practiced in wildlife areas, may increase the threat of fires, with negative impact on the environment and biodiversity.

HPAI is a disease of poultry that evolves from milder viral strains naturally occurring within wild bird populations (13). Hence the hunters that raise poultry and practice birds cleaning at home, could favor low pathogenic AIVs evolve to HPAI (49). Backyard poultry have played different roles in AI epidemics across affected countries (50, 51). Nonetheless, is desirable to prevent backyard poultry exposure to AIVs. Despite the low epidemic potential of AIV infection in backyard poultry, for many families in developing countries, poultry are more than a source of income or food but also play social and cultural roles. Hence backyard poultry must be preserved.

The feeding of domestic animals with birds' leftover could lead to an increase in the host range of the virus and even the disease, as well as the emergence of new subtypes due to the phenomenon of genetic reassortment. It has been shown that antigenic and genetic evolution of IAVs often results in inter-species transmission as the virus adapts to a new host (52). In fact, reports of influenza virus affecting dogs (53, 54) are relatively recent, but they have been important in causing epidemic outbreaks mainly in greyhounds (55). On the other hand, pigs are susceptible to IAVs of avian and human origin (56, 57), which may cause the emergence of new virus. In fact, the H1N1 pandemic virus in 2009 resulted from a novel reassortant among avian, human and swine origin viruses (58, 59).

In the current study, the use of dogs for hunting did not prevent contact of hunters with water. The persistence of AIVs in water and their fecal-oral transmission among waterbirds are of recognized importance in the maintenance of the virus in the ecosystem (60). Therefore, water contact for hunters may result in their exposure to AIVs. Conversely, there are not records of AIV infection in humans acquired through water, despite this material and sediments in wetlands being an important source of such viruses (49).

Direct contact with water during hunting should be a practice to avoid because in addition to the threat of AIVs some other severe disease-causing pathogens like leptospira may be present in wetlands. Interestingly the recruited hunters for the present study indicated higher levels of vaccination coverage for leptospirosis compared to seasonal flu (results no showed).

Higher levels of hunter confidence due to more years of experience could reduce risk perception due to usual practices that apparently do not affect their health, as observed in other studies (10). The risk of infection by AIVs demands the development of communication strategies that improve knowledge through dissemination of public health messages that may cause a change in behavior among hunters. A sound knowledge of the potential risk factors that facilitate the introduction and spread of AIVs in animal and human populations is key to developing preventive control strategies and contributing to active surveillance programs.

No taking into consideration the variables with the inferior limit of OR<1, only smoking and having backyard birds at home remained as significant according to Cerda (61). In particular, smoking habit encompass a well-known health risk itself (48), but it seems not enough to withdraw such practice. On the other hand, having backyard poultry at home it is not a risk, if that are not exposed to AIVs through practices like cleaning hunted birds at home which had OR <1 even at the superior limit of CI 95%.

### Study Strengths and Limitations

Our study aimed to gain an understanding of the bird harvesting practices and attitudes regarding AI exposure among Cuban hunters and to identify gaps in influenza pandemic plans. This research provides information on the population strata (hunters) that have more influence on the risk of infection and dissemination of AIVs. It complements the anti-pandemic plan in the face of the possibility of infections with this pathogen in humans, bearing in mind the necessity of contact between animals and people as a prerequisite for this to occur (8). In addition, it contributes to the strategy's improvement for managing the risk of introduction and dissemination of the AIVs in Cuba. Poultry production in Cuba is an important component of livestock economy with over 35.35 million heads (including hens, ducks, turkeys, quails, among others) with their own breeders (62). The main production from the commercial poultry sector are eggs with a consumption average over 200 per capita egg/year, hence it is an important component of food security.

The location of hunter groups in geographically different areas did not allow for random sampling because a representative group of people is hard to be matched in time and space. Almost 1% of the registered hunters in Cuba were recruited for the study, although active hunting could vary with the availability of cartridges and transportation to hunting sites.

### Conclusions

Cuban hunters participate in some practices while harvesting wild birds that could potentially expose them and their domestic animals to AIVs. There was no relation between protective measures reported by hunters and their awareness on avian influenza, which may imply a lack of knowledge on AIV. This study emphasizes the need to introduce more effective risk communication strategies about the consequences of AIVs infecting humans or other animals and emphasizes the importance of reducing risks and exposure.

## Data Availability Statement

The raw data supporting the conclusions of this article will be made available by the authors, without undue reservation.

## Ethics Statement

The studies involving human participants were reviewed and approved by Research Ethical Committee of the National Center for Animal and Plant Health (CENSA). The patients/participants provided their written informed consent to participate in this study.

## Author Contributions

PA, LM, and MA: conceptualization and design of the study. BD-H, FP, DM, and JA: data collection. BD-H, PA, LM, and YA: data processing, analyses, and interpretation. MA and MP: formal analysis. BD-H, PA, and LM: writing—original draft preparation. PA, LM, and MP: writing—review and editing. All authors have read and agreed to the published version of the manuscript.

## Conflict of Interest

The authors declare that the research was conducted in the absence of any commercial or financial relationships that could be construed as a potential conflict of interest.

## Publisher's Note

All claims expressed in this article are solely those of the authors and do not necessarily represent those of their affiliated organizations, or those of the publisher, the editors and the reviewers. Any product that may be evaluated in this article, or claim that may be made by its manufacturer, is not guaranteed or endorsed by the publisher.
