# Supplementary material for: Knowledge, Attitudes, and Risk Perception Toward Avian Influenza Virus Exposure Among Cuban Hunters
Source: Front Public Health. 2021 Jul 23;9:644786. doi: 10.3389/fpubh.2021.644786 (PMC8342762; doi:10.3389/fpubh.2021.644786)
Supplement: Supplementary file 1 [file Data_Sheet_1.PDF]

frontiers

Supplementary information

Table S1. Risk analyses by variables according to hunter hierarchic status within the Federation (Hunters belonging to FCCD Steering Committee versus those only dedicated to hunting)

Variable	B	S.E.	Wald	P	OR [95% CI for OR]
Feed domestic animal with birds leftover	0.586	0.313	3.518	0.061	1.798 [0.974 - 3.318]
Having backyard birds at home	0.673	0.298	5.097	0.024	1.961 [1.093 - 3.517]
Unvaccinated against flu	-0.842	0.347	5.903	0.015	0.431 [0.218 - 0.850]
Smoking	0.858	0.347	6.119	0.013	2.357 [1.195 - 4.650]
Don´t Cleaning knives after hunting	1.426	0.612	5.423	0.020	4.163 [1.253 - 13.827]
Sharing hunting knives with household uses	0.342	0.294	1.351	0.245	1.407 [0.791 - 2.503]
Don´t washing hands during hunting	1.402	0.613	5.239	0.022	4.065 [1.223 - 13.510]
Cleaning hunted birds at home	-1.116	0.424	6.942	0.008	0.327 [0.143 - 0.751]
Get assistance for birds cleaning	0.101	0.290	0.121	0.728	1.106 [0.627 - 1.952]
Hunting with dogs	-0.649	0.308	4.437	0.035	0.523 [0.286 - 0.956]
Water contact during hunting	-0.484	0.374	1.678	0.195	0.616 [0.296 - 1.282]
Wild bird hunting as risk for health	-0.272	0.299	0.832	0.362	0.761 [0.424 - 1.367]
Knowledge about avian influenza	-1.699	0.535	10.096	0.001	0.183 [0.064 - 0.522]

B: estimated slope: S.E.: standard error; OR: Odds ratio[Exp(B)]

Table S2. Squared correlation (eta2) between the variables and the respective dimension for hunter´s exposure to avian influenza virus

Variable	Dim 1	Dim 2	Dim 3	Dim 4
HuntDog	0.000	0.159	0.194	0.339
WaterContact	0.060	0.321	0.000	0.000
BirdCleanHome	0.109	0.332	0.001	0.006
BirdCleanAssist	0.008	0.144	0.419	0.001
DontCleanHands	0.206	0.085	0.230	0.028
ShareHuntKnives	0.000	0.100	0.015	0.676
DontKniveClean	0.263	0.059	0.181	0.004
Smoker	0.544	0.154	0.127	0.000
FluUnvaccinated	0.601	0.126	0.073	0.006
Variability (%)	19.899	36.335	50.121	61.899

HuntDog: Hunt with dog; WaterContact: Direct contact with water; BirdCleanHome: Cleaning birds at home; BirdCleanAssist: Receiving help for bird cleaning; DontCleanHands: Don´t washing hands; ShareHuntingKnives: Sharing hunting knives in domestic activities; DontKniveClean: Don´t cleaning knives; FluUnvaccinated: Not be vaccinated against flu.

Table S3. Squared correlation (eta2) between the variables and the respective dimension for domestic animal exposure to avian influenza virus

**Variable**	**Dim 1**	**Dim 2**
HuntDog	0.564	0.001
BirdCleanHome	0.092	0.830
PoultryHome	0.565	0.019
FeedAnimals	0.303	0.131
Variability (%)	38.106	62.661

HuntDog: Hunt with dog; BirdCleanHome: Cleaning birds at home; PoultryHome: Having backyard birds at home; *FeedAnimals: Feed domestic animal with bird leftover.*

SURVEY

This questionnaire is intended to identify capacity building needs for people who practice activities related to bird hunting. The personal data requested are of demographic interest and the information will be handled anonymously. Your willingness to fill out the questionnaire is highly appreciated as an expression of your agreement to allow the use of the information in a scientific study.

Age ________ Gender: M ___ F ___ Years spent hunting ______
Approximate number of days hunting per year ______
Approximate number of birds hunted per year _______
Mark the months in which you hunt:

Jan	Feb	March	April	May	Jun	Jul	Aug	Sep	Oct	Nov	Dic

List in order, from highest to lowest, the five species of birds more frequently hunted:____
____
____
Do you have direct contact with water when hunting? YES __ NO __
Is there any hazard to your health for handling wild birds? YES __ NO __
Do you clean hunted birds in the field? YES __ NO __
Do you take the hunted birds to your home for cleaning sometimes? YES _ NO_
Do you get help from someone for bird cleaning? YES __ NO__
Do you wash your hands immediately after cleaning birds? YES __ NO __ and do you use a disinfectant? YES __ NO __
Are your hunting knives used for other foods? YES __ NO __
Do you clean knives and other instruments every time you hunt? YES __ NO __
Do you smoke? YES __ NO __
What do you do with the bird's leftover after bird cleaning?
____
Do you practice hunting assisted by dogs? YES __ NO __
Do you raise chickens in your home? YES __ NO__
Do you feed other animals with wild birds leftover? YES __ NO __
If yes, indicate: dogs ___ pigs ___ chickens ___ others ___
Do you know what is the avian influenza or avian flu? YES __ NO__
Point out how you hunt birds: in flight YES __ NO __, being on land or in water YES __ NO __, captures birds with moving difficulties YES __ NO __
Have you been vaccinated against human influenza? YES __ NO __, if yes, please state the last year of vaccination _______
Have you been vaccinated against leptospirosis YES __ NO __, if yes, state the last year of vaccination _______
